# An Intensive ^18^F-Fludeoxyglucose–Positron Emission Tomography With Computed Tomography–Based Strategy of Follow-Up in Patients Treated for Head and Neck Squamous Cell Carcinoma Who Are Clinically Asymptomatic

**DOI:** 10.1001/jamanetworkopen.2023.26654

**Published:** 2023-08-01

**Authors:** Jean-Christophe Leclère, Camille Clément, Romain Le Pennec, Clementine Maheo, Dorothy M. Gujral, Ulrike Schick, Grégoire Le Gal, Remi Marianowski, Pierre-Yves Salaun, Ronan Abgral

**Affiliations:** 1 Head and Neck Surgery Department, University Hospital of Brest, Brest, France; 2 Nuclear Medicine Department, University Hospital of Brest, Brest, France; 3 UMR Inserm 1304 GETBO, University of Western Brittany, Brest, France; 4 Clinical Oncology Department, Imperial College Healthcare National Health Service Trust, Charing Cross Hospital, London, United Kingdom; 5 Department of Cancer and Surgery, Imperial College London, London, United Kingdom; 6 Radiotherapy Department, University Hospital of Brest, Brest, France; 7 Clinical Investigation Center CIC 1412, University Hospital of Brest, Brest, France

## Abstract

**Importance:**

Patients with head and neck squamous cell carcinoma (HNSCC) have a significant risk of locoregional recurrence within the first 2 years, with approximately two-thirds of patients experiencing such recurrence. While early recurrence detection may be associated with improved patient outcomes, the association of such detection with survival remains uncertain.

**Objective:**

To investigate the association of an intensive posttreatment follow-up strategy using ^18^F-fludeoxyglucose–positron emission tomography with computed tomography (^18^FDG-PET/CT) with survival among patients with HNSCC.

**Design, Setting, and Participants:**

This case-control study was conducted among patients treated at 1 of 3 locations in Brest, France (University Hospital, Military Hospital, or Pasteur Clinic). The statistical analysis was conducted from January to June 2023. All adults with histologically proven HNSCC who were treated with curative intent between January 1, 2006, and December 31, 2019, and achieved a complete response on imaging at 3 to 6 months were included. They had a minimum of 3 years of follow-up.

**Exposures:**

Patients undergoing an intensive posttreatment follow-up strategy had ^18^FDG-PET/CT (PET/CT group) at months 12, 24, and 36, chosen at the discretion of ear, nose, and throat surgeons.

**Main Outcomes and Measures:**

Overall survival (OS) at 3 years.

**Results:**

Among 782 patients with HNSCC (642 males [82.1%]; median [IQR] age, 61 [56-68] years), 497 patients had ^18^FDG-PET/CT during follow-up and 285 patients had conventional follow-up (CFU group). Cox regression analysis showed an association between undergoing ^18^FDG-PET/CT and lower risk of death (odds ratio, 0.71; 95% CI, 0.57-0.88; *P* = .002) after adjustment for covariates (age, sex, comorbidities, primary location, stage, surgeon, year of treatment, and treatment). The mean (SD) 3-year OS was significantly better in the PET/CT vs CFU group (72.5% [2.0%] vs 64.3% [2.9%]; *P* = .002). Analysis based on American Joint Committee on Cancer stage showed significantly better mean (SD) 3-year OS for advanced stages III and IV in the PET/CT group (373 patients) vs CFU group (180 patients; 68.5% [2.4%] vs 55.4% [3.8%]; *P* < .001), while no significant difference was observed between patients with stage I or II HNSCC. Analysis based on primary tumor site revealed significantly longer mean (SD) 3-year OS for oropharyngeal tumor in the PET/CT group (176 patients) than the CFU group (100 patients; 69.9% [3.5%] vs 60.5% [5.0%]; *P* = .04).

**Conclusions and relevance:**

This case-control study found that use of ^18^FDG-PET/CT in the standard annual CFU of HNSCC was associated with a 3-year survival benefit, with a larger benefit for patients with advanced initial tumor stage (III-IV) and oropharyngeal disease.

## Key Points

**Question:**

Is an intensive follow-up strategy based on ^18^F-fludeoxyglucose–positron emission tomography with computed tomography (^18^FDG-PET/CT) associated with survival in patients treated for head and neck squamous cell carcinoma (HNSCC) who are clinically asymptomatic?

**Findings:**

In this case-control study that included 782 adults with HNSCC, mean 3-year overall survival was better in the PET/CT group (72.5%) than the conventional follow-up group (64.3%), a significant difference.

**Meaning:**

This study found that use of ^18^FDG-PET/CT in the annual standard follow-up of HNSCC was associated with a 3-year survival benefit.

## Teaser

This case-series study investigates the association between intensive ^18^F-fludeoxyglucose–positron emission tomography with computed tomography (^18^FDG-PET/CT)–based follow-up and survival in patients with clinically asymptomatic head and neck squamous cell carcinoma.

## Introduction

Head and neck squamous cell carcinomas (HNSCCs) are the sixth most common malignant tumor worldwide, with approximately 800 000 new cases annually.^1^ HNSCCs encompass cancers of the oral cavity, oropharynx, hypopharynx, and larynx and are traditionally linked to tobacco and alcohol addiction and human papillomavirus positive status.^2^ Despite recent advances in treatment regimens and modalities, patients have a poor prognosis, particularly because there is a high rate of locoregional recurrences during the first 2 years in two-thirds of cases.^3^ Posttreatment surveillance of HNSCC is therefore crucial and raises many diagnostic challenges.

The conventional follow-up (CFU) according to guidelines currently relies on iterative clinical examination in the first 3 years after treatment and cervical computed tomography (CT), magnetic resonance imaging (MRI), or both within 3 to 6 months after treatment to define a new radiological baseline. This is done given that patient anatomy is frequently altered after surgical resection, complex reconstructive surgery, and adjuvant radiation.^4,5^ Currently, no routine morphological cervical imaging is recommended for patients who are asymptomatic during surveillance except an annual chest CT, which is particularly encouraged in ongoing smokers.^6^

Use of ^18^F-fluorodesoxyglucose–positron emission tomography with computed tomography (^18^FDG-PET/CT) is currently recommended in the posttreatment setting if recurrence is clinically suspected and for staging to look for distant metastasis if locoregional recurrence is confirmed.^6,7,8^ Studies^9,10^ have already shown the high performance of ^18^FDG-PET/CT in diagnosing subclinical disease recurrence during systematic follow-up. A 2015 meta-analysis^11^ conducted on 7 studies with a total of 907 patients found a 14.2% detection rate of occult relapse, with pooled sensitivity and specificity of 89% and 92%, respectively. Advantages of ^18^FDG-PET/CT vs conventional imaging include its higher performance in differentiating posttreatment tissue distortion from locoregional recurrence.^12,13,14^ In addition, it can reveal distant relapse or metachronous primary cancer (MPC) in a single whole-body scan without significantly increasing radiation levels compared with dedicated morphological neck imaging.^15,16^

To date, the use of such an intensive posttreatment follow-up strategy using ^18^FDG-PET/CT is still optional and focused on patients with locally advanced disease at diagnosis.^8^ Indeed, its usefulness remains controversial,^17^ notably because of the debate on its cost-effectiveness,^18,19^ lack of consensus on schedule of monitoring, and paucity of data on prognostic outcomes associated with ^18^FDG-PET/CT monitoring. These outcomes have been suggested in a few small series, although results of 2 ongoing multicenter randomized prospective trials may answer these issues.^20,21^ The aim of this study was therefore to investigate the association of an intensive strategy of posttreatment follow-up using ^18^FDG-PET/CT with survival vs standard CFU (including chest CT) in a large retrospective case-control cohort of patients treated for HNSCC.

## Methods

This case-control study was approved by the Institutional Ethics Committee at Brest University Hospital, and all patients provided written informed consent. The study followed the French General Data Protection Regulation. We followed the Strengthening the Reporting of Observational Studies in Epidemiology (STROBE) reporting guideline for the writing of this study.

### Population

Patients aged 18 years or older with newly diagnosed and histologically proven HNSCC between January 1, 2006, and December 31, 2019, achieving a complete imaging response at 3 to 6 months after curative intent treatment and being followed up in the University Hospital, Military Hospital, or Pasteur Clinic of Brest, France, were retrospectively included. Patients with residual disease after primary treatment were excluded.

Several patient characteristics at the time of the multidisciplinary team meeting were collected: body mass index (BMI; calculated as weight in kilograms divided by height in meters squared), cancer history, other primary HNSCC history, immunodeficiency, performance status, smoking, alcohol consumption, primary tumor location, tumor stage, and synchronous cancer. Tumors were staged according to the American Joint Committee on Cancer Classification (AJCC) *AJCC Cancer Staging Manual* seventh and eighth editions within the inclusion period.^22,23^

### Follow-Up

Patients were followed up for at least 3 years with conventional CFU according to published guidelines,^6^ including clinical examination every 2 months during the first year, 3 months during the second year, and 4 months during the third year. Surveillance lung imaging at months 12, 24, and 36 (M12, M24, and M36) with a chest CT (CFU group) or ^18^FDG-PET/CT (PET/CT group) was chosen at the discretion of ear nose and throat surgeons.

### Imaging

Examinations were performed during the study period on several successive PET/CT hybrid machines as follows: between 2006 to 2012 on a Gemini GXLi (Philips Healthcare) system and from 2012 to 2019 on a Biograph-mCT (Siemens ) system. After intravenous injection of 3 to 5 MBq/kg of ^18^FDG (IBA Molecular Imaging), patients were encouraged to remain calm and at rest (staying bedbound for approximately 1 hour).

CT was initially performed in the craniocaudal direction, with a whole-body protocol and injection of iodine contrast (1.5 mL/kg) after confirmation of no contraindication to contrast. Whole-body PET/CT data were acquired in 3D mode and included emission images (2 to 3 minutes per step) and transmission images required for attenuation correction. Transmission images were obtained from x-ray scan data. Emission images were corrected for background noise and random events and reconstructed with and without attenuation correction using the iterative line of response row-action maximum likelihood algorithm method for the Gemini system and the iterative ordered subset expectation maximization (with point spread function modeling and time-of-flight acquisition capabilities) method for the Biograph system. PET images were smoothed with a Gaussian filter (full width at half maximum = 2 mm). The 6-slice Gemini and 40-slice Biograph scanners had 600-mm and 700-mm transverse fields of view, respectively.

### Statistical Analysis

The primary end point for this study was overall survival (OS), defined as the time from diagnosis to death from any cause. Patients lost to follow-up were censored during the survival analysis. The Kaplan-Meier method was used to estimate OS. We used the log-rank test to compare 3-year OS rates between CFU and PET/CT groups in the whole cohort and based on AJCC stage or primary tumor location. A Cox regression model was used to assess the association of PET/CT addition with survival outcomes. This Cox model allowed us to analyze time-to-event data, with survival as the primary outcome of interest. By including PET/CT as an explanatory variable in the model, its association with survival was examined while adjusting for other relevant covariates, such as age (per 1-year increase), male sex (reference group: female), cancer history, immunodeficiency, World Health Organization performance status score 0 or 1 (reference group: 2 or 3), smoking (>10 pack-years), alcohol (>3 drinks/d), primary location (5 locations: oral cavity [primary reference], oropharynx, larynx, hypopharynx, and carcinoma unknown primary), early stage I to II (reference group: advanced stage III-IV), surgeon (15 surgeons; primary reference: Louis Bonne, MD [Brest Military Hospital]) (eAppendix in Supplement 1), first curative-intent treatment surgery (reference group: radiotherapy), and year of treatment (before median [range] year of 2014 [2006-2019] vs on or after median year [reference]). Cox model assumptions were checked by graphical inspection of Schoenfeld residuals plotted against time. The Fisher exact test and Mann-Whitney test were used to compare qualitative and quantitative variables between groups. The level of significance was *P* < .05, and statistical tests were 2-sided. Statistical analyses were performed using SPSS statistical software version 25 (IBM). The statistical analysis was conducted from January to June 2023.

## Results

### Population

Among 782 patients (642 males [82.1%]; median [IQR; range] age, 61 [56-68; 32-95] years), there were 497 patients in the PET/CT group and 285 patients in the CFU group. Clinical characteristics are available in Table 1. Two-thirds of the entire cohort (569 patients [72.7%]) were treated at the university hospital, and 42 patients (5.4%) had a previous history of HNSCC from another primary location. The most common tumor site was the oropharynx (276 patients [35.3%]), and carcinoma of unknown primary tumor accounted for 21 cases (2.7%). The distribution of main curative intent treatments (surgery and radiotherapy) was balanced (391 patients [50.0%] vs 391 patients [50.0%]). A total of 47 patients (6.0%), including 27 patients (5.4%) in the PET/CT group and 20 patients (7.0%) in the CFU group, were lost to follow-up at 3 years.

**Table 1.** Patient Characteristics

Characteristic	No. (%)			*P* value
	PET/CT group (n = 497)	CFU group (n = 285)	Total (N = 782)	
Age, mean (SD), y	62 (8)	62 (8)	62 (8)	.72
BMI, mean (SD)	23.6 (3.8)	23.5 (3.2)	23.5 (3.7)	.21
Sex				
Male	404 (81.3)	238 (83.5)	642 (82.1)	.50
Female	93 (18.7)	47 (16.5)	140 (17.9)	
Cancer history	123 (24.7)	38 (13.3)	161 (20.6)	.002
HNSCC history	30 (6.0)	12 (5.0)	42 (5.4)	.32
Immunodeficiency	43 (8.7)	9 (3.2)	52 (6.6)	.003
Performance status				
0	289 (58.1)	208 (73.0)	497 (63.6)	<.001
1	164 (33.0)	64 (22.5)	228 (29.2)	.002
2	38 (7.6)	9 (3.2)	47 (6.0)	.01
3	6 (1.2)	4 (1.4)	10 (1.3)	1
Smoking >10 pack-years	462 (93)	273 (95.8)	735 (94.0)	.12
Alcohol >3 drinks/d	194 (39.0)	76 (26.7)	270 (34.5)	<.001
Primary tumor location				
Oral cavity	106 (21.3)	63 (22.1)	169 (21.6)	.86
Oropharynx	176 (35.4)	100 (35.1)	276 (35.3)	.94
HPV positive	38 (21.6)	12 (12.0)	50 (18.1)	.05
HPV unknown	109 (61.9)	76 (76.0)	185 (67.0)	.02
Larynx	122 (24.5)	72 (25.3)	194 (24.8)	.86
Hypopharynx	75 (15.1)	46 (16.1)	121 (15.5)	.76
CUP	18 (3.6)	4 (1.1)	21 (2.7)	.04
AJCC stage				
Early stage (I-II)	124 (24.9)	103 (36.1)	227 (29.0)	<.001
I	75 (15.1)	64 (22.5)	139 (17.8)	.01
II	49 (9.9)	39 (13.7)	88 (11.3)	.13
Advanced stage (III-IV)	373 (75.1)	180 (63.2)	553 (70.7)	<.001
III	84 (17.3)	37 (13.0)	121 (15.5)	.15
IV	289 (58.1)	143 (50.2)	432 (55.2)	.04
Synchronous cancer	31 (6.2)	13 (4.6)	44 (5.6)	.42
Treatment				
Surgery	237 (47.7)	154 (53.7)	391 (50.0)	.12
Alone	96 (19.3)	72 (25.3)	168 (21.5)	.06
With RT	86 (17.3)	47 (16.5)	133 (16.9)	.84
With CRT	55 (11.1)	35 (12.3)	90 (11.5)	.64
Radiotherapy	260 (52.3)	131 (46.3)	391 (50.0)	.10
Alone	74 (14.9)	27 (9.5)	101 (13.0)	.04
With CT	186 (37.4)	104 (36.5)	290 (37.1)	.82
Lost to follow-up at 3 y	27 (5.4)	20 (7.0)	47 (6.0)	.43

Abbreviations: AJCC, American Joint Committee on Cancer; BMI, body mass index (calculated as weight in kilograms divided by height in meters squared); CFU, conventional follow-up; CRT, chemoradiotherapy; CT, chemotherapy; CUP, carcinoma unknown primary; HNSCC, head and neck squamous cell carcinoma; HPV, human papillomavirus; PET, positron emission tomography; RT, radiotherapy.

Several patient clinical characteristics at diagnosis were significantly worse in the PET/CT vs CFU group. The PET/CT group had higher prevalence of cancer history (123 patients [24.7%] vs 38 patients [13.3%]; *P* = .002), more patients with immunodeficiency (43 patients [8.7%] vs 9 patients [3.2%]; *P* = .003), fewer patients with a performance status score of 0 (289 patients [58.1%] vs 208 patients [73.0%]; *P* < .001), higher rates of alcohol consumption of more than 3 drinks per day (194 patients [39.0%] vs 76 patients [26.7%]; *P* < .001), and more patients with an advanced stage (AJCC stages III-IV; 373 patients [75.1%] vs 180 patients [63.2%]; *P* < .001). Regardless of the treatment received, 123 of 164 recurrences (75.0%) occurred within 660 days after treatment.

### Use of ^18^FDG-PET/CT

Patients in the PET/CT group underwent ^18^FDG-PET/CT at M12 (497 patients [100%]), M24 (370 patients [74.4%]), and M36 (289 patients [58.1%]). During the first 3 years of follow-up, 111 relapses (22.3%), of which 62 relapses (55.9%) were detected by annual ^18^FDG-PET/CT in patients who were clinically asymptomatic. The overall detection rate of subclinical recurrence by ^18^FDG-PET/CT was 62 patients (12.4%). Of the total subclinical recurrences, 36 recurrences (58.1%), 19 recurrences (30.6%), and 7 recurrences (11.3%) were detected by ^18^FDG-PET/CT at M12, M24, and M36, respectively. These occurred in primary tumors of the oropharynx (27 patients), larynx (15 patients), hypopharynx (10 patients), and oral cavity (8 patients); 2 patients had carcinoma unknown primary. Annual ^18^FDG-PET/CT revealed 51 MPCs (10.3%), including 19 lung cancers (37.3%; 3.8% of patients in the PET/CT group) and 12 other primary head and neck cancers (23.5%; 2.4% of patients in the PET/CT group). Including these MPCs, there were 113 subclinical events overall (22.7%). Excluding pulmonary and esophageal metachronous disease, 28 MPCs (54.9%) detected by ^18^FDG-PET/CT were extrathoracic.

### Survival

**Figure 1.**
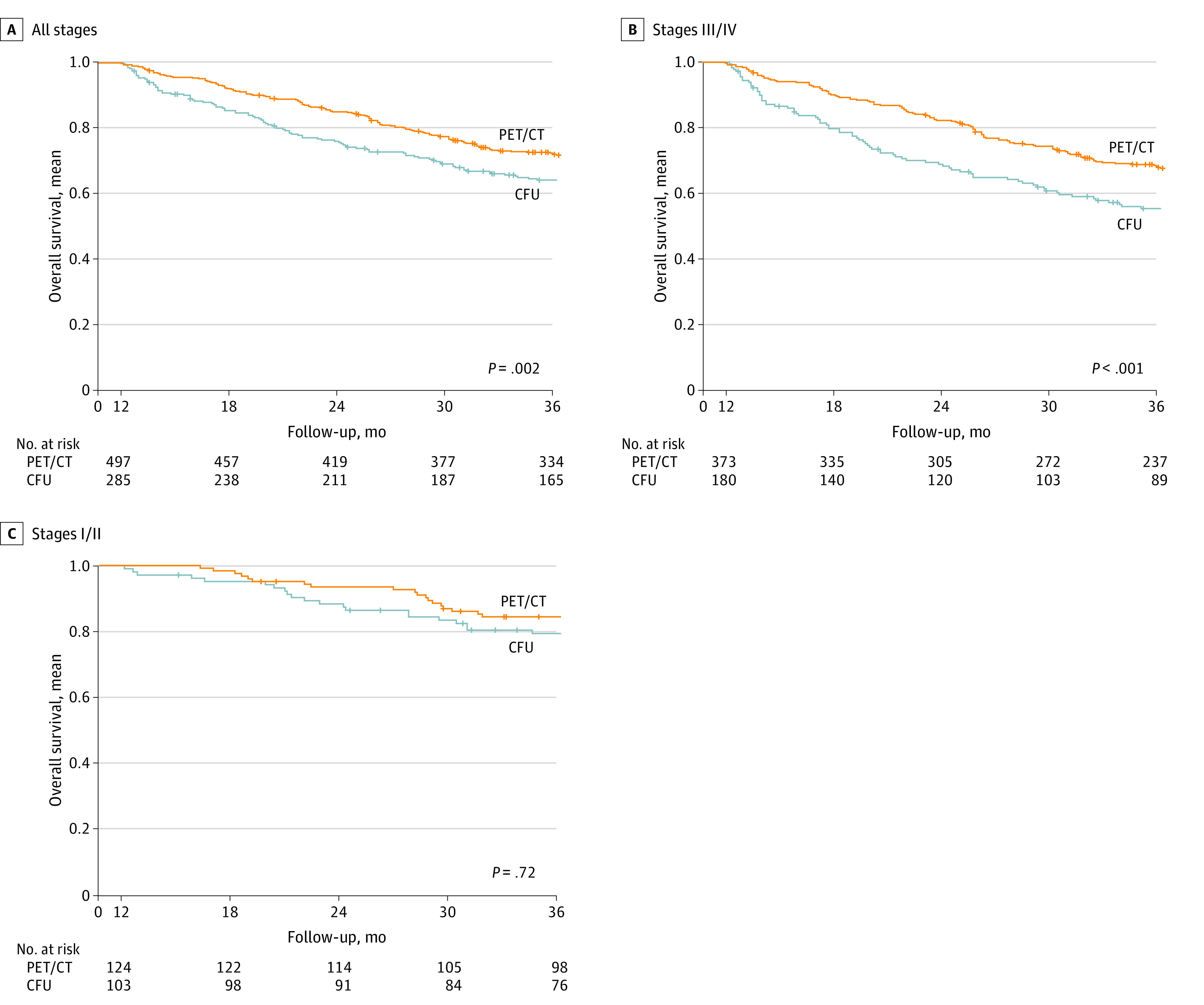
Survival at 3 Years by Cancer Stage Kaplan-Meier curves of 3-year overall survival are presented in positron emission tomography with computed tomography (PET/CT) and conventional follow-up (CFU) groups by American Joint Committee on Cancer stage. A, All stages (782 patients). B, Advanced stages III and IV (553 patients). C, Early stages I and II (227 patients). Plus signs indicate censored individuals.

**Figure 2.**
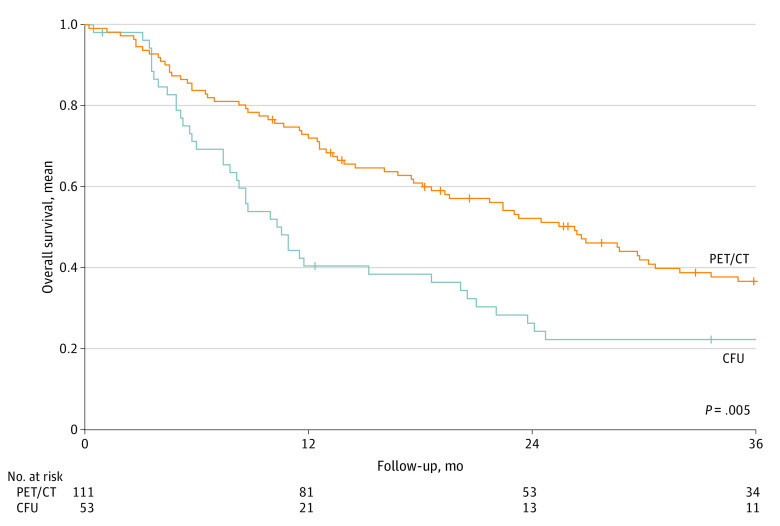
Survival at 3 Years After Recurrence Detection Kaplan-Meier curves of 3-year overall survival after recurrence detection are presented in positron emission 19 tomography with computed tomography (PET/CT) and conventional follow-up (CFU) groups. Plus signs indicate censored individuals.

The mean (SD) 3-year OS was significantly better in the PET/CT vs CFU group (72.5% [2.0%] vs 64.3% [2.9%]; *P* = .002) regardless of the initial stage at diagnosis (Figure 1A). Cox regression analysis showed an association between undergoing ^18^FDG-PET/CT and lower risk of death (odds ratio, 0.71; 95% CI, 0.57-0.88; *P* = .002) compared with CFU after adjustment for covariates (age, sex, comorbidities, primary location, stage, surgeon, year of treatment, and treatment) (Table 2). For recurrent disease (164 patients), mean (SD) 3-year OS was significantly better in the PET/CT group vs the CFU group (36.4% [3.8%] vs 22.2% [4.6%]; *P* = .005) (Figure 2).

**Table 2.** Cox Analysis With Survival as Primary Outcome^a^

Factor	OR (95% CI)	*P* value
PET/CT during follow-up	0.71 (0.57-0.88)	.002
Covariate		
Male sex	1.35 (1.01-1.80)	.45
Age, y (per 1-year increase)	1.02 (1.01-1.03)	.001
Cancer history	1.55 (1.18-2.02)	.001
Immunodeficiency	1.14 (0.75-1.74)	.53
Performance status 0 or 1 (reference: 2 or 3)	0.58 (0.41-0.82)	.002
Smoking >10 pack-years	1.34 (0.84-2.141)	.22
Alcohol >3 drinks/d	1.20 (0.97-1.48)	.10
Early stage I-II (reference: III-IV)	0.66 (0.51-0.85)	.001
Primary location (5 locations)^b^	NA	.56
Surgeon (15 surgeons)^c^	NA	.14
Year of treatment before median in 2014 (reference: on or after median)	1.60 (1.25-2.04)	<.001
First curative-intent treatment surgery (reference group: radiotherapy)	0.67 (0.53-0.84)	<.001

Abbreviations: CT, computed tomography; NA, not applicable; OR, odds ratio; PET, positron emission tomography.

^a^ In this analysis, PET/CT was included as an explanatory variable, while other parameters served as covariates.

^b^ The 5 locations were oral cavity (primary reference), oropharynx, larynx, hypopharynx, and carcinoma unknown primary.

^c^ See the eAppendix in Supplement 1 for 15 surgeons; primary reference: Louis Bonne, MD (Brest Military Hospital).

#### AJCC Stage

There was a longer mean (SD) 3-year OS for III to IV advanced stages in the PET/CT group (373 patients) than the CFU group (180 patients; 68.5% [2.4%] vs 55.4% [3.8%]; *P* < .001) (Figure 1B). There was no difference in mean (SD) 3-year OS between stage I to II in the PET/CT group (124 patients) vs CFU group (103 patients; 84.5% [3.3%] vs 79.4% [4.0%]; *P* = .72) (Figure 1C).

#### Primary Tumor Location

**Figure 3.**
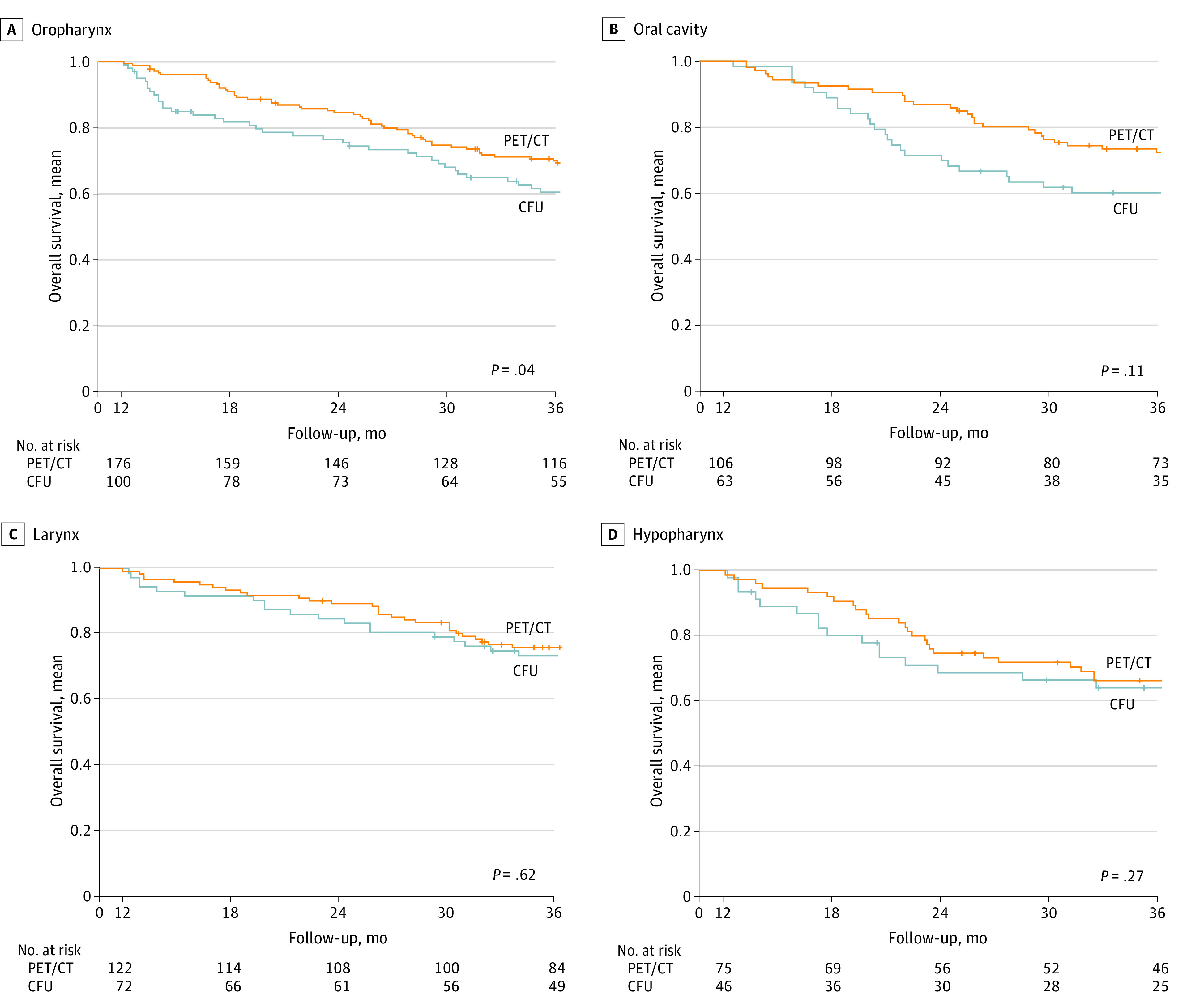
Survival at 3 Years by Primary Tumor Location Kaplan-Meier curves of 3-year overall survival are presented in positron emission tomography with computed tomography (PET/CT) and conventional follow-up (CFU) groups by primary tumor location. A, Oropharynx (276 patients). B, Oral cavity (169 patients). C, Larynx (194 patients). D, Hypopharynx (121 patients). Plus signs indicate censored individuals.

There was a significantly longer mean (SD) 3-year OS for oropharyngeal tumors in the PET/CT group (176 patients) compared with the CFU group (100 patients; 69.9% [3.5%] vs 60.5% [5.0%]; *P* = .04) (Figure 3A). There was no statistically significant difference in mean (SD) 3-year OS for oral cavity tumors in the PET/CT group (106 patients) vs the CFU group (63 patients; 72.4% [4.4%] vs 60.1% [6.2%]; *P* = .11) (Figure 3B). There was no difference in mean (SD) 3-year OS between the PET/CT and CFU groups with laryngeal (75.9 [3.9%] vs 73.4% [5.2%]; *P* = .62) or hypopharyngeal (66.3% [5.5%] vs 64.1% [7.2%]; *P* = .27) tumors (Figure 3C and D). There were 91 subclinical recurrences (81.9%) and 46 metachronous cancers (90.2%) ultimately treated with curative intent.

## Discussion

This case-control study found that integration of ^18^FDG-PET/CT in the posttreatment follow-up of patients with HNSCC was associated with a significantly improved mean (SD) 3-year OS compared with standard monitoring (72.5% [2.0%] vs 64.3% [2.9%]; *P* = .002). To the best of our knowledge, this is the first series demonstrating a significant survival difference in patients monitored in an intensive follow-up strategy based on ^18^FDG-PET/CT. In a retrospective study^24^ including 257 patients treated for HNSCC, ^18^FDG-PET/CT detected clinically occult recurrence in 9% of patients at 12 months and 4% of patients at 24 months. However, no difference in outcomes was identified between PET/CT-detected and clinically detected recurrences, with similar 3-year disease-free survival (41% vs 46%; *P* = .91) and 3-year OS (60% vs 54%; *P* = .70). Our study found that after recurrent disease (164 patients), mean (SD) 3-year OS in the PET/CT group was significantly better than in the CFU group (36.4% [3.8%] vs 22.2% [4.6%]; *P* = .005). Moreover, Kim et al^25^ concluded that in their study of 153 patients with HNSCC, positive PET/CT findings at 3, 6, and 12 months after treatment were associated with poor OS. However, it is important to note that they did not compare these results with a control group of patients without PET monitoring.

First, we hypothesize that this difference in survival in our study may be associated with the 12.4% overall detection rate of subclinical recurrence in the PET/CT group, which was similar to that reported in the literature. In a meta-analysis of 7 studies that included 907 patients, Sheikhbahaei et al^11^ found a similar overall detection rate of occult recurrences (14%). We also found a decrease in subclinical recurrence detection rate by ^18^FDG-PET/CT with time after treatment (36 recurrences at M12, 19 recurrences at M24, and 7 recurrences at M36), similar to that reported by Ho et al.^24^ By including 51 metachronous cancers diagnosed with ^18^FDG-PET/CT, our overall rate of subclinical events was 22.7%. This was in accordance with the rates reported by the retrospective study undertaken by Dunsky et al,^26^ who reported 24 asymptomatic lesions (recurrent or new primary lesions; 20%) detected by ^18^FDG-PET/CT in 123 patients treated for HNSCC who were asymptomatic.

In our subgroup analyses, patients diagnosed with advanced-stage disease had a better mean (SD) 3-year OS in the PET/CT group (68.5% [2.4%] vs 55.4% [3.8%]; *P* < .001), unlike patients with early stage cancer. Several series have already shown that patients with III to IV AJCC advanced stages were at higher risk of asymptomatic recurrences and advocated ^18^FDG-PET/CT as an option for surveillance of these patients, in accordance with published guidelines.^8,11^ Our results highlighted improved mean (SD) 3-year OS in the PET/CT vs CFU group for patients with oropharyngeal cancers (69.9% [3.5%] vs 60.5% [5.0%]; *P* = .04), which was the most common tumor location in our cohort, in keeping with published data.^1^ For other primary tumor locations, the lack of difference may be explained by the insufficient statistical power in subgroups. This interesting finding may open the discussion to include PET/CT imaging in the monitoring of oropharyngeal tumors.

In our cohort, 75% of recurrences occurred within 660 days after treatment, regardless of treatment received. This is consistent with published data, suggesting the usefulness of PET/CT monitoring in the first 2 years after treatment. In a retrospective study of 388 patients who underwent sequential PET/CT scanning every 3 months after treatment, Beswick et al^27^ found no benefit associated with PET/CT imaging after 2 years. Indeed, they reported that 95% of recurrences occurred within 24 months after treatment, with a significant proportion (66%) diagnosed by ^18^FDG-PET/CT in patients who were asymptomatic. Moreover, in a retrospective series of 324 oral cavity SCC cases with complete surgical resection followed by ^18^FDG-PET/CT at 3 to 6 months and 1 year, Fukomoto et al^28^ found that 75% of recurrences occurred within 400 days. They reported that recurrences were detected clinically at a significantly later time compared with those detected by ^18^FDG-PET/CT (mean time, 839.3 vs 345.1 days; *P* = .001). Finally, Kim et al^25^ retrospectively reported 17.5% occult recurrences detected by ^18^FDG-PET/CT at M12 after treatment, compared with 7.2% in our PET/CT group. This difference may be partly explained by the combined analysis of occult recurrence and metachronous cancer in their study.

Another explanation for the difference in OS in our series may be the 10.3% rate of MPCs reported in the PET/CT group. Most of these were in the pulmonary and head and neck areas. These results are similar to those reported in the literature. The overall reported incidence of MPCs in patients treated for HNSCC ranged from 8.1% to 16.3% in some series,^29,30^ and the rate per year remained constant during the follow-up period.^31^ In addition, Liu et al^32^ found that the main sites of MPCs were the head and neck area, esophagus, and lung in a cohort of 5914 patients treated for pharyngolaryngeal cancers. In a meta-analysis of 26 studies, Hoxhaj et al^33^ found similar results in terms of sites for MPCs in 72 450 patients, primarily associated with the same risk factors (alcohol and tobacco consumption). Among PET/CT studies, Krabbe et al^34^ reported a 6.7% rate of lung MPCs in a cohort of 149 patients with HNSCC who were asymptomatic compared with 3.8% in our study. Moreover, in our previous published prospective series, we reported an overall MPC detection rate of 7 of 91 patients (7.7%)^9^ and 6 of 116 patients (5.1%)^10^ using ^18^FDG-PET/CT in patients who were clinically asymptomatic. This difference may be explained by the difficulty in differentiating a single lung metastasis from MPC with SCC histology. Finally, after excluding pulmonary and esophageal metachronous disease, 28 MPCs detected by 18FDG-PET/CT were extra-thoracic (54.9%) and therefore not located in the field of view of a chest CT. If we assume a similar proportion of extrathoracic MPCs in our CFU group, this may be associated with patient outcome, as 90.2% of MPCs were treated with curative intent in the PET/CT group.

### Limitations

This study has several limitations. First, the retrospective, single-center design requires validation by multicenter randomized prospective studies to limit selection bias. There are 2 such trials currently underway.^20,21^ Second, in our case-control study, there was a greater number of patients in the PET/CT group than the CFU group, and the choice of follow-up modalities was at the discretion of the ear, nose, and throat surgeon. This difference may be explained by changes in the routine practice of most clinicians over the years, given that we have demonstrated in previous prospective studies^9,10^ the high performance of ^18^FDG-PET/CT in the detection of subclinical recurrences. These data were also incorporated in this analysis.^9,10^ Third, patient characteristics were more unfavorable in the PET/CT group, which could have been associated with the difference in survival between groups. Fourth, patients were not clinically examined on the same day as ^18^FDG-PET/CT was performed, which may potentially contribute to an increased rate of subclinical recurrences. Nevertheless, we specified that the time between the last negative clinical examination and the PET/CT had to always be less than 1 month to limit this risk of overestimation. Fifth, the long period of inclusion (13 years) saw a notable evolution in treatment methods and medical imaging techniques, but the median year of patient treatment was similar in both groups (2014). Sixth, our follow-up period was limited to 3 years. Most recurrent events were diagnosed over the first 2 years. Given this, the optimal follow-up schedule and duration remain to be determined. Seventh, there were no available data for human papilloma virus status in two-thirds of patients because this was not recommended during the first years of the inclusion period.

## Conclusions

This case-control study found that use of ^18^FDG-PET/CT as an alternative to annual chest CT in the follow-up of HNSCC was associated with incremental 3-year OS benefit, specifically in patients with advanced disease at diagnosis (stage III-IV) or oropharyngeal primary tumors. Prospective multicenter randomized studies are needed to investigate a casual relationship with survival and may help to define a follow-up schedule.

## Notes

Supplement 1. **eAppendix.** List of Surgeons

Supplement 2.
Data Sharing Statement
