# Supplementary material for: An Intensive 18F-Fludeoxyglucose–Positron Emission Tomography With Computed Tomography–Based Strategy of Follow-Up in Patients Treated for Head and Neck Squamous Cell Carcinoma Who Are Clinically Asymptomatic
Source: JAMA Netw Open. 2023 Aug 1;6(8):e2326654. doi: 10.1001/jamanetworkopen.2023.26654 (PMC10394574; doi:10.1001/jamanetworkopen.2023.26654)
Supplement: Supplement 1. — eAppendix. List of Surgeons [file jamanetwopen-e2326654-s001.pdf]

Supplemental Online Content

Leclère JC, Clément C, Le Pennec R, et al. An intensive ^{18}F-fludeoxyglucose–positron emission tomography with computed tomography–based strategy of follow-up in patients treated for head and neck squamous cell carcinoma who are clinically asymptomatic. *JAMA Netw Open*. 2023;6(8):e2326654. doi:10.1001/jamanetworkopen.2023.26654

eAppendix. List of Surgeons

This supplemental material has been provided by the authors to give readers additional information about their work.

© 2023 Leclère JC et al. *JAMA Network Open.*

eAppendix. List of Surgeons

Dr Louis Bonne, Dr Coralie Clodic, Dr Paul Corbeau, Dr Benoit Feger, Dr Yves Gauvin, Dr Yves Gobel, Dr Jean-Christophe Leclère, Dr Marie-Suzanne Le Gac, Pr Rémi Marianowski, Dr Julien Prévot, Dr Gaël Potard, Dr Bertrand Ravary, Dr Florence Rogez, Dr Pierre-Yves Vaillant, Dr Gérald Valette.

Primary reference: Dr Louis Bonne.

© 2023 Leclère JC et al. *JAMA Network Open.*
