# Supplementary material for: An Intensive 18F-Fludeoxyglucose–Positron Emission Tomography With Computed Tomography–Based Strategy of Follow-Up in Patients Treated for Head and Neck Squamous Cell Carcinoma Who Are Clinically Asymptomatic
Source: JAMA Netw Open. 2023 Aug 1;6(8):e2326654. doi: 10.1001/jamanetworkopen.2023.26654 (PMC10394574; doi:10.1001/jamanetworkopen.2023.26654)
Supplement: Supplement 2. — Data Sharing Statement [file jamanetwopen-e2326654-s002.pdf]

Data Sharing Statement

Leclère. An Intensive ^{18}F-Fludeoxyglucose–Positron Emission Tomography With Computed Tomography–Based Strategy of Follow-Up in Patients Treated for Head and Neck Squamous Cell Carcinoma Who Are Clinically Asymptomatic. *JAMA Netw Open*. Published online August 1, 2023. doi:10.1001/jamanetworkopen.2023.26654

DataData available: No

Additional Information
Explanation for why data not available: if request
